# Microbial community dynamics and coexistence in a sulfide-driven phototrophic bloom

**DOI:** 10.1186/s40793-019-0348-0

**Published:** 2020-01-17

**Authors:** Srijak Bhatnagar, Elise S. Cowley, Sebastian H. Kopf, Sherlynette Pérez Castro, Sean Kearney, Scott C. Dawson, Kurt Hanselmann, S. Emil Ruff

**Affiliations:** 1 Department of Biological Sciences, University of Calgary, Calgary, AB Canada; 2 School of Medicine and Public Health, University of Wisconsin-Madison, Madison, WI USA; 3 Department of Geological Sciences, University of Colorado, Boulder, CO USA; 4 Ecosystems Center and J. Bay Paul Center for Comparative Molecular Biology and Evolution, Marine Biological Laboratory, Woods Hole, MA USA; 5 Department of Biological Engineering, Massachusetts Institute of Technology, Cambridge, MA USA; 6 Department of Microbiology and Molecular Genetics, University of California Davis, Davis, CA USA; 7 Department of Earth Sciences, ETH, Zürich, Switzerland

**Keywords:** Microbial succession, Green sulfur bacteria, Prosthecochloris, Syntrophy, Brackish coastal ecosystem, Anoxygenic phototrophy, Myoviridae, Sulfur cycling, CRISPR-Cas, Resilience

## Abstract

**Background:**

Lagoons are common along coastlines worldwide and are important for biogeochemical element cycling, coastal biodiversity, coastal erosion protection and blue carbon sequestration. These ecosystems are frequently disturbed by weather, tides, and human activities. Here, we investigated a shallow lagoon in New England. The brackish ecosystem releases hydrogen sulfide particularly upon physical disturbance, causing blooms of anoxygenic sulfur-oxidizing phototrophs. To study the habitat, microbial community structure, assembly and function we carried out in situ experiments investigating the bloom dynamics over time.

**Results:**

Phototrophic microbial mats and permanently or seasonally stratified water columns commonly contain multiple phototrophic lineages that coexist based on their light, oxygen and nutrient preferences. We describe similar coexistence patterns and ecological niches in estuarine planktonic blooms of phototrophs. The water column showed steep gradients of oxygen, pH, sulfate, sulfide, and salinity. The upper part of the bloom was dominated by aerobic phototrophic *Cyanobacteria*, the middle and lower parts by anoxygenic purple sulfur bacteria (*Chromatiales*) and green sulfur bacteria (*Chlorobiales*), respectively. We show stable coexistence of phototrophic lineages from five bacterial phyla and present metagenome-assembled genomes (MAGs) of two uncultured *Chlorobaculum* and *Prosthecochloris* species. In addition to genes involved in sulfur oxidation and photopigment biosynthesis the MAGs contained complete operons encoding for terminal oxidases. The metagenomes also contained numerous contigs affiliating with *Myoviridae* viruses, potentially affecting *Chlorobi*. Our data suggest a short sulfur cycle within the bloom in which elemental sulfur produced by sulfide-oxidizing phototrophs is most likely reduced back to sulfide by *Desulfuromonas sp*.

**Conclusions:**

The release of sulfide creates a habitat selecting for anoxygenic sulfur-oxidizing phototrophs, which in turn create a niche for sulfur reducers. Strong syntrophism between these guilds apparently drives a short sulfur cycle that may explain the rapid development of the bloom. The fast growth and high biomass yield of *Chlorobi*-affiliated organisms implies that the studied lineages of green sulfur bacteria can thrive in hypoxic habitats. This oxygen tolerance is corroborated by oxidases found in MAGs of uncultured *Chlorobi*. The findings improve our understanding of the ecology and ecophysiology of anoxygenic phototrophs and their impact on the coupled biogeochemical cycles of sulfur and carbon.

## Background

Estuarine and coastal water bodies are dynamic and widespread ecosystems that are often characterized by the mixing of terrestrial freshwater and ocean saltwater. The resulting brackish habitats have physical and chemical characteristics that differ from those found in fresh and saltwater ecosystems [1, 2]. Brackish ecosystems are often very productive and support rich microbial and macrobial communities [1]. Estuaries provide crucial ecosystem services, the most salient of which are trapping and filtering terrestrial runoffs and pollutants before they reach the oceans, coastal protection, erosion control and habitat-fishery linkages [3–6].

Estuaries harbor abundant and diverse microbial communities that are part of a complex food web. Autotrophic microbes fix carbon dioxide through photosynthesis or chemosynthesis [7–9], while heterotrophs remineralize carbon introduced to estuaries as organic matter from the oceans or land [10–12]. The decomposition of sulfur containing organic compounds through fermentation can lead to the production of sulfide in estuarine sediments [13]. Furthermore, sulfate from seawater can be reduced by sulfate respirers to elemental sulfur or sulfide [13, 14]. Sulfate introduced by the ocean and sulfide released from the sediments form gradients in the water column that cause the development of a chemocline [15]. Additionally, estuaries and coastal marshes often exhibit a halocline, i.e. a change in salinity, and the depletion of oxygen in the water column can create an oxycline [16, 17]. Overlapping gradients, e.g. in salinity, light availability, as well as oxygen and sulfide concentration create habitats and niches that favor certain microbial communities and conversely microbial communities can affect and respond to such gradients [18–20].

Gradients of oxygen and sulfur compounds in stratified aquatic environments as well as the penetration depth of radiation offer conditions for the development of complex and stable microbial assemblages [21]. These gradients are usually divided into a surface layer rich in oxygen, an intermediate layer with decreasing oxygen and a bottom anoxic layer. The surface layer is often dominated by oxygenic phototrophic microorganism such as *Cyanobacteria* and algae. The anoxic layer, particularly in systems with high organic loads, provides niches for anaerobes such as sulfate-reducing bacteria [22]. In the intermediate layer, anoxygenic phototrophs use the light from the surface and the sulfide from the bottom layers [23]. The biogeochemical processes leading to stratification in phototrophic blooms are relatively well understood [24], yet ecological niches, microbial interactions and community dynamics are less well constrained.

The abiotic and biotic drivers of stratified estuarine environments can fluctuate frequently and rapidly as a result of tidal cycles, weather events, and seasonal cycles [25–30]. Such fluctuations can cause noticeable changes in the microbial community structure of an ecosystem. It has been shown that estuarine communities are structured by salinity [31–34], precipitation [32, 35], temperature [33, 34], oxygen [35, 36] and also seasonal changes [34]. Community shifts included changes in phytoplankton populations with salinity [31], declining populations of *Rhodobacterales* with decreasing salinity [35], declining populations of phototrophic "*Candidatus* Aquiluna" with decreasing oxygen concentration, as well as general changes in the richness and evenness of the community [31–36].

**Fig. 1:**
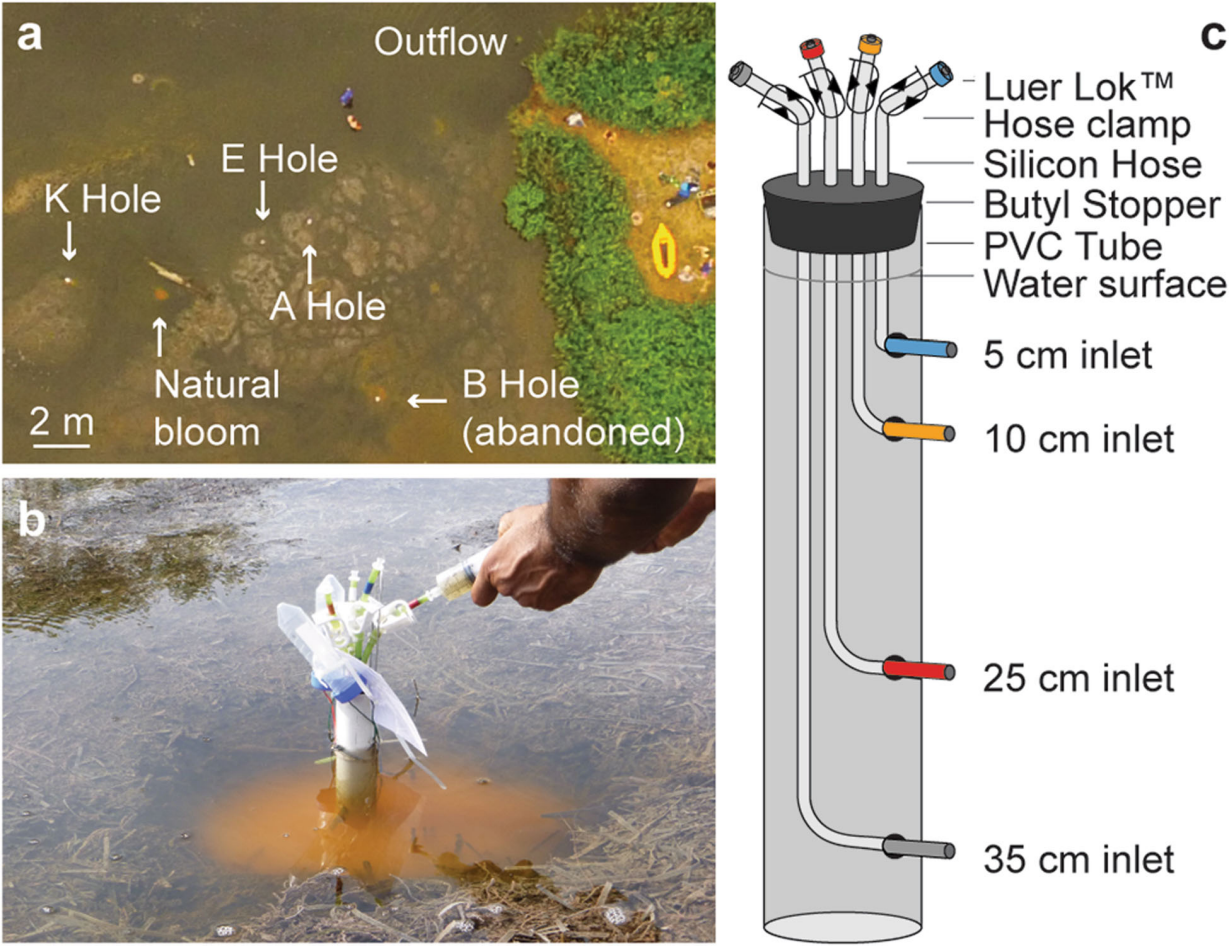
Sampling sites. **a** Aerial view of experimental sites (A, E, and K) in the Trunk River lagoon. The water enters the lagoon from the left and exits to the sea through a channel marked outflow. **b** Phototrophic bloom and sampling pole during sample collection at time point 3; 5 days post-disturbance. **c** Schematic of a sampling pole

Trunk River lagoon in Falmouth, MA, is a brackish ecosystem, on the coast of Vineyard Sound (N 41.535236, W − 70.641298). Storms, tides, and run-off introduce large amounts of biomass forming thick layers of decaying seagrass and other organic matter. The lagoon has a sulfidic odor and emanates gases formed in the organic matter deposits. Bright yellow microbial blooms can be observed occasionally just below the water surface (see Fig. 1, Additional file 1: Figure S1), forming and disappearing within days to weeks. Transient blooms were observed to occur in natural depressions in the decaying organic matter and were apparently initiated by physical disturbance events, potentially from storms, tidal extremes, human activity, or animals. Given this natural ecological progression, we tested whether experimentally induced physical disturbance could trigger bloom formation, and whether the established blooms could be used as a model system to investigate the microbial ecology and ecophysiology of sulfur-oxidizing phototrophs.

We mimicked physical disturbances of the brackish ecosystem by creating artificial depressions in the decaying organic matter, and monitored the microbial community response and population dynamics, as well as ecological niches of the key populations. Based on the above described observations of thick layers of decaying organic matter and naturally occurring, rapid blooms of phototrophs, we hypothesize that i) the disturbance would release sulfide from the sediment and cause a sulfide-driven phototrophic bloom, ii) due to its rapid development the bloom would likely be dominated by very few populations, and iii) steep physicochemical gradients would establish creating (transient) anoxic habitats in the water column analogous to blooms in stratified lakes. The remarkably reproducible community assembly and succession provides insights into niches and coexistence of phototrophic microorganisms in a small scale ecosystem. Our findings contribute to the understanding of the ecological processes and dynamics in phototrophic blooms, which are naturally occurring phenomena in many ecosystems.

## Results

This study was designed to investigate microbial community assembly, community turnover and syntrophic interactions in a sulfide-driven phototrophic bloom. To gain insights into the microorganisms niches and potential key metabolisms we studied the physicochemistry of the water column, the diversity of photopigments, and performed amplicon and metagenomic sequencing.

### Physicochemistry of the water column

**Fig. 2:**
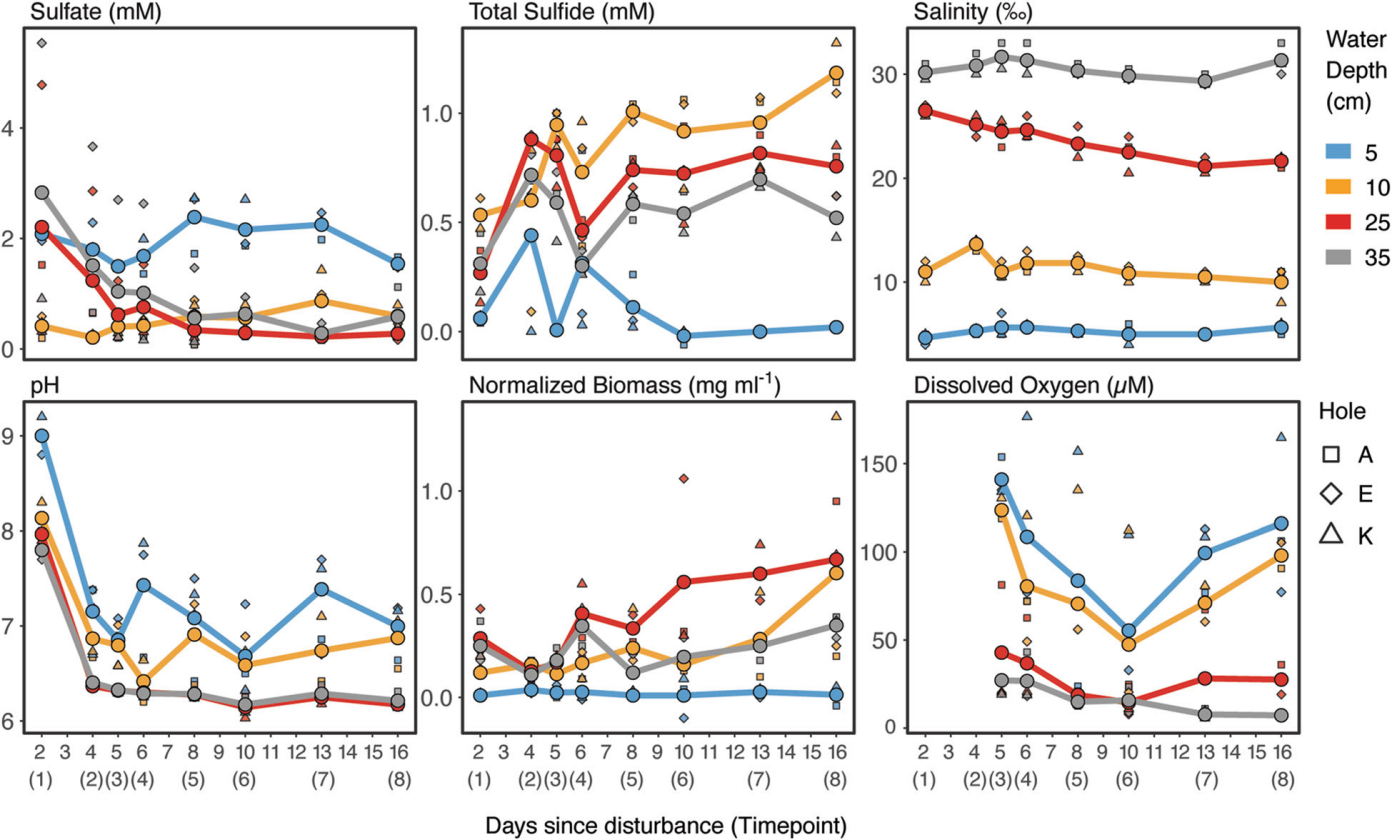
Physicochemical measurements at the sampling sites. Measurements are shown as averages (circles) across the three replicate holes. Measurements at individual holes are shown as squares, diamonds and triangles, the trend is shown as lines connecting average values. The x-axis shows days since disturbance and sampling timepoints in brackets. The y-axis shows the respective units. For an alternative representation of the physicochemical parameters as depth profiles instead of temporal profiles, see Additional file 1: Figure S5. For further parameters (Fe (II); Fe (III); Total Fe, nitrate) refer to Additional file 1: Figure S6

At the first sampling time point (two days post-disturbance), no difference in color was observed in the water column. Two days later, a faint pink layer was observed in the water column, and faint shades of yellow appeared in samples from 25 cm depth (Additional file 1: Figure S2, Supplementing Results). The yellow color of the suspension was most intense from timepoint 4 to 7 and had almost disappeared by timepoint 8. Within the first three days of the experiment the pH decreased between one and two units in all layers, with lowest values present in the deepest layer (Fig. 2). Over the 15-day sampling period, pH showed more variation in the two upper layers than in the two deeper layers where it was very constant at values between pH 6 to 6.3. Throughout the experiment the water column in all three experiments had a stable halocline with brackish water (5 ‰ salinity) at the water surface and saltwater (30 ‰) at 35 cm depth (Fig. 2). Salinity increased with depth and was 12 ‰ and 23 ‰ at 10 cm and 25 cm, respectively. Major ions also reflect this trend (e.g. calcium, potassium in Additional file 1: Figure S6). The dissolved oxygen (DO) concentrations showed a relatively stable oxycline between 10 and 25 cm. At 10 cm and above, DO was mostly higher than 50 μM (91 ± 45 μM) corresponding to ~ 20 % oxygen saturation (36 ± 17 %). At 25 and 35 cm DO was mostly below 50 μM (23 ± 18 μM), hence below ~ 20 % (9 ± 9 %) saturation. The oxygen concentration slowly decreased in the upper two layers during the first half of the experiment but recovered to the initial values towards the end of the experiment. At 5 and 10 cm, DO averaged over the experiment was 101 ± 47 μM and 81 ± 41 μM, respectively (Fig. 2). At 25 and 35 cm, the average DO was 28 ± 22 μM and 17 ± 11 μM, respectively. The sulfate concentrations in the water column decreased along the depth gradient, with the highest sulfate concentration at 5 cm (≈ 2 mM) and the lowest at 25 cm (≈ 0.2 mM) (Fig. 2). In contrast, the sulfide concentrations were lowest at 5 cm (Fig. 2f). Interestingly, the greatest sulfide concentration was measured at 10 cm depth peaking at over 1 mM towards the end of the experiment. Below 10 cm, sulfide concentration was still high, but declined to 0.75 mM ± 0.22 at 25 cm and 0.5 mM ± 0.17 at 35 cm. The normalized biomass measured for the 5 cm samples throughout the sampling period was nearly zero (Fig. 2). At 10 cm, 25 cm, and 35 cm, the normalized biomass measured was approximately, 0.2, 0.3, and 0.2 mg ml^-1^, respectively. For details concerning iron (Fe(II), Fe(III), total Fe), nitrate, calcium, potassium, ammonium and acetate refer to Additional file 1: Supplementary Results and Figure S6. Overall, the measurements revealed stable and reproducible physicochemical gradients that divided the previously homogenous water column into layers with different redox conditions and energy availability.

### Spectral absorbance of phototrophic community

**Fig. 3:**
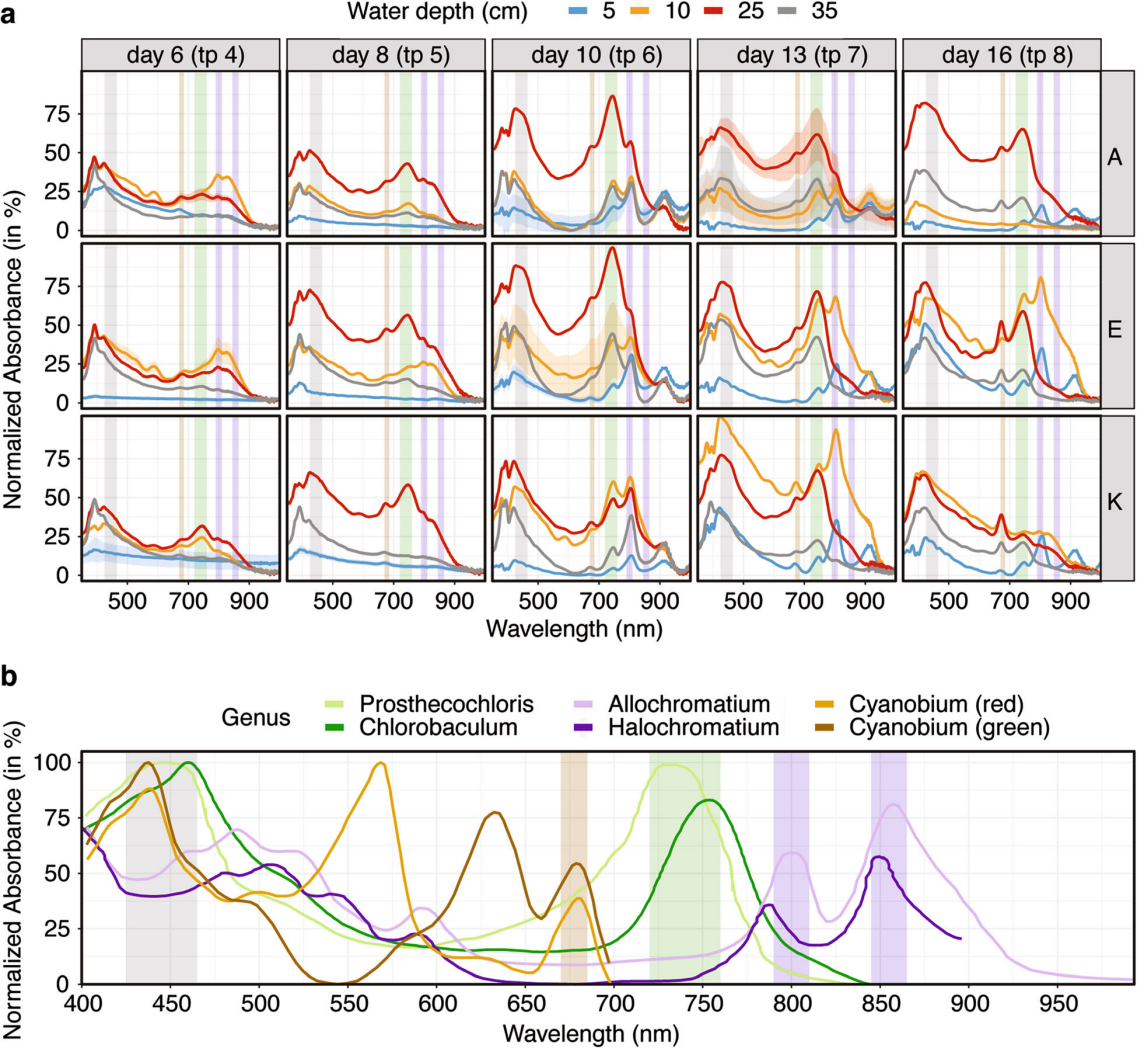
Spectral Absorbance. **a** Sample spectra for each depth at the three sites and five different time points. Each spectrum represents the average of at least three replicate spectral analyses per sample. Confidence bands along the spectra indicate standard deviation (bands are mostly smaller than the center line and thus not visible). Green and purple vertical bands indicate major absorbance peaks of photopigments characteristic for the GSB group (*Prosthecochloris* and *Chlorobaculum*, 720–760 nm) and the PSB group (*Allochromatium* and *Halochromatium*, 790–810 nm and 845–865 nm), respectively, highlighting the transient appearance, succession and overall importance of these anoxygenic phototrophs over the course of the experiments. Also indicated is the general phototroph absorbance peak at 425–465 nm as a light gray vertical band. Cyanobacterial photopigments have distinct absorbance peaks in the 500–700 nm range that are not prominent in the sample spectra except for the characteristic 670–685 nm peak (light brown vertical band) reflecting the presence but likely minor role of these taxa during the experiment. **b** Photopigment absorbance spectra from pure culture representatives of major phototrophic lineages. Vertical bands are, as in panel A, highlighting diagnostic absorbance peaks of GSB (in green), PSB (in purple), Cyanobacteria (in brown) and phototrophs in general (in gray). All absorbance spectra were normalized to the respective highest peak

We measured absorbance spectra from filters of samples from experiment A, E and K (Fig. 3a) and compared the spectra to those of representative cultured species of phototrophic genera from the literature [37–41] (Fig. 3b). Our results suggest that pigments belonging to PSB, indicated by purple vertical bands (Fig. 3a), were abundant in the upper layer of the bloom (orange spectra in Fig. 3a) especially between day 10 and 13. GSB pigments, indicated by a green vertical band, dominated the lower layers of the bloom (red and gray spectra) starting at day 10. Pigments characteristic for *Cyanobacteria* (brown vertical band) were less abundant in the bloom but increased at the end of the experiment relative to the PSB and GSB peaks. This suggests a minor role of *Cyanobacteria* initially and during the bloom but a more important role upon return to equilibrium. Pigments present in all major phototrophic lineages were detected throughout the experiment (gray vertical band). The results of the spectral analysis suggest the coexistence of multiple phototrophic lineages over the entire duration of the experiment.

### Microbial community structure and taxonomic composition

**Fig. 4:**
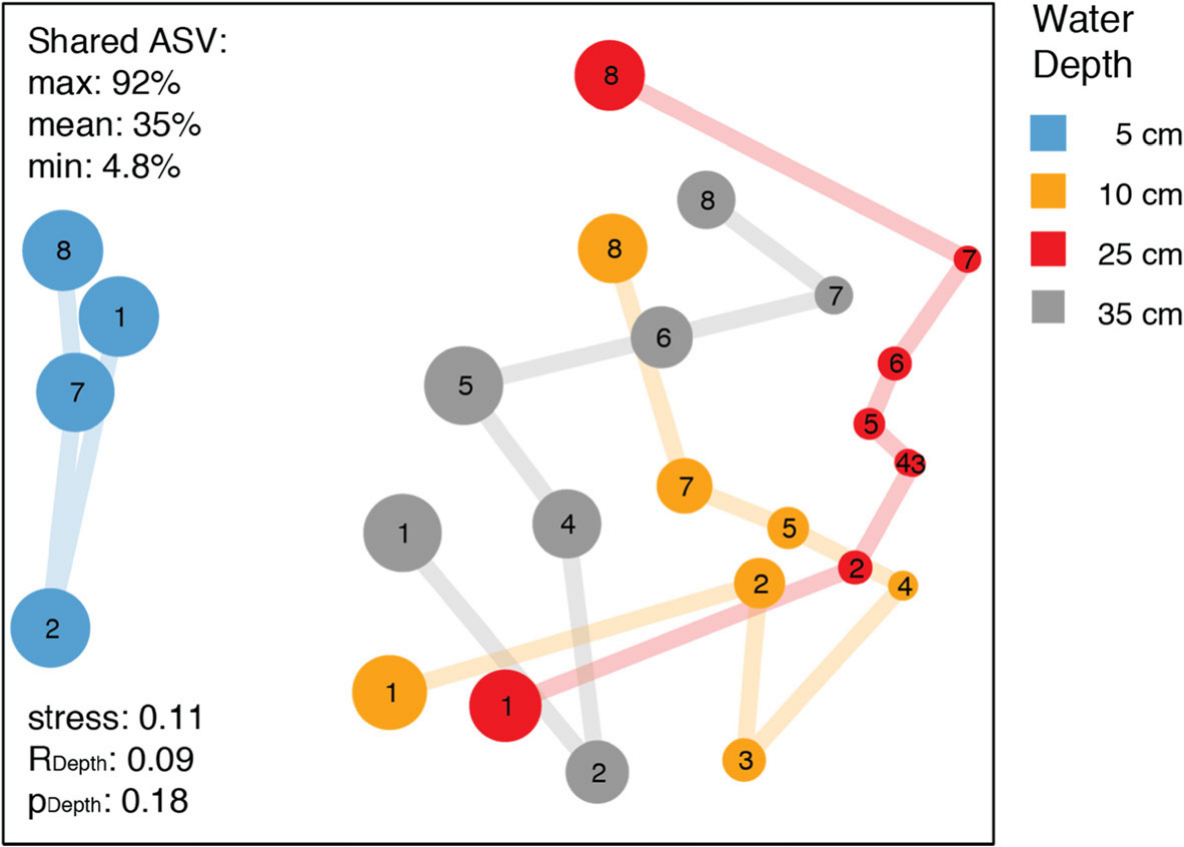
Microbial Community Turnover. Non-metric multidimensional scaling (NMDS) ordination based on relative abundance of ASVs (amplicon sequence variants). Each circle represents one sample, the closer two samples are the more similar is their microbial community structure. Circle size represents Shannon Diversity. Numbers indicate sampling time points. Colors indicate bloom layers. Note: Individual holes were very similar (see Additional file 1: Figure S9A) and thus we averaged relative ASV abundances for clarity, i.e. each circle represents an average across replicate experiments. NMDS ordinations for individual experiments are shown in Additional file 1: Figure S8. The communities in the different layers of individual experiments are significantly different, yet overlapping (see Additional file 1: Figure S8)

**Fig. 5:**
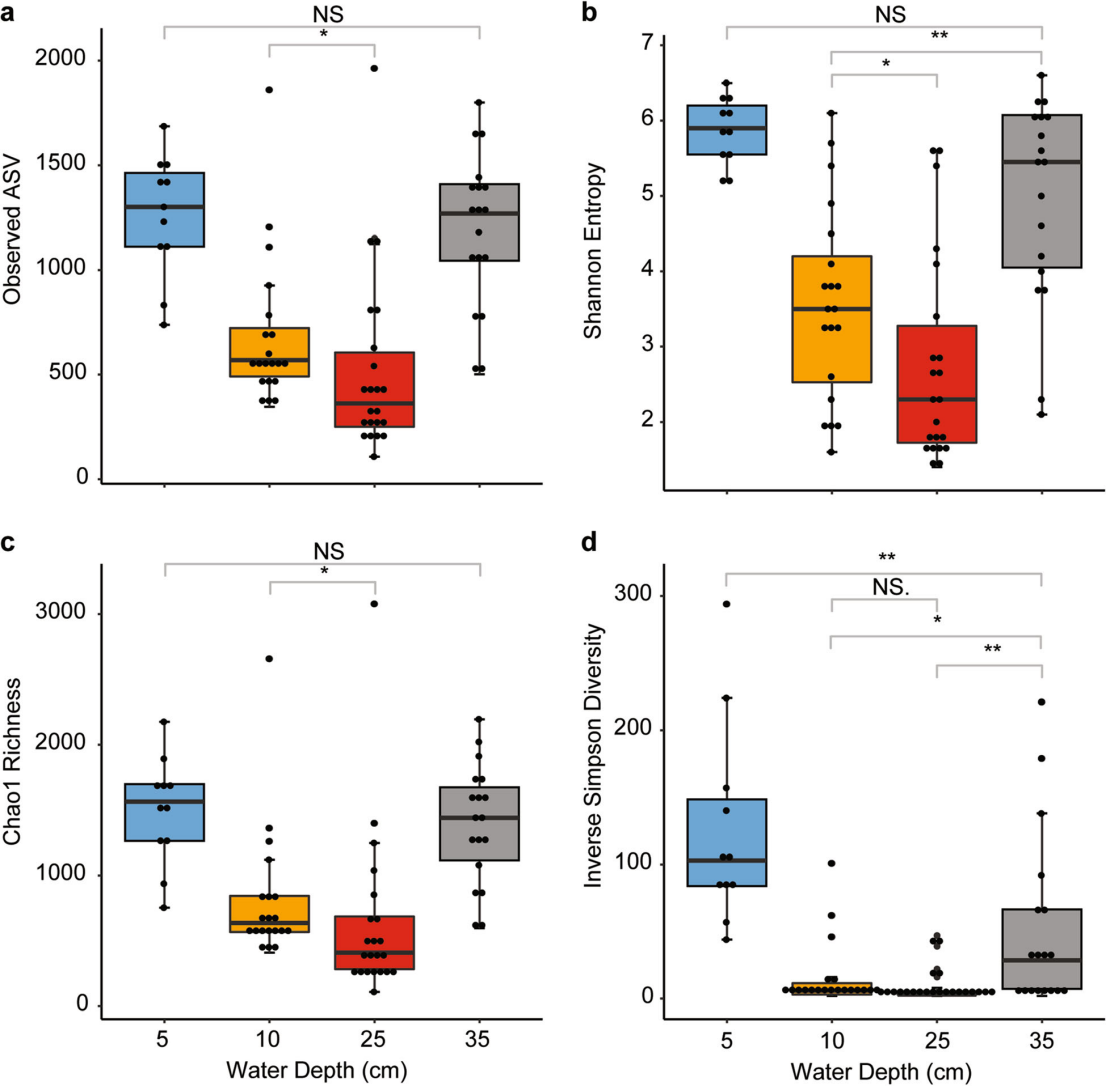
Diversity Indices of all samples grouped by depth. **a** Richness is shown as the number of observed amplicon sequence variants (ASVs). Richness gives equal weight to all ASVs regardless of their abundance. **b** Shannon entropy considers ASV richness and proportional abundance, **c** Chao1 richness represents an estimate of the total number of ASVs that may be present in the sample.** d** Inverse Simpson diversity considers ASV richness and proportional abundance, similar to Shannon entropy, but gives additional weight to proportional abundance (evenness). All indices show significantly lower diversity in the bloom layers, especially at 25 cm depth, as compared to the top and bottom layers. Diversity indices were calculated using a subsampling approach to account for unequal sampling effort. Pairwise comparisons with low significance levels are shown (NS, *: *p* < 0.1, **: *p* < 0.01). All pairwise comparisons that are not shown were highly significant (***: *p* < 0.001), e.g. panel a 5 cm vs 10 cm

At the beginning of the experiment, the microbial diversity was high in all four water depths and very similar across replicate ecosystems. Alpha diversity rapidly decreased with the onset of the bloom, and within two days the communities in the four depth layers substantially changed (Figs. 4 and 5, Additional file 1: Figure S7, S8). The bloom occurred between 10 and 25 cm water depth (Additional file 1: Figure S2) with highest cell numbers (peaking at > 10^8^ cells ml^− 1^) and biomass at around 25 cm water depth (Fig. 2, Additional file 1: Figure S4) in brackish, mildly acidic, and hypoxic waters (Fig. 2). The number of observed amplicon sequence variants (ASVs), as well as estimated richness, Shannon entropy, and Inverse Simpson diversity significantly decreased between the surface water and the water at a depth of 10 cm and 25 cm (Fig. 5; *p* = 0.001). This change is most striking in the case of Inverse Simpson diversity, a measure for evenness. In just 1 day, evenness dropped in both 10 cm and 25 cm water depth by over one order of magnitude to low single digit values (Additional file 1: Table S1). This means the community was dominated by one ASV (a pure culture has an Inverse Simpson diversity index of 1). This decrease in diversity was accompanied by a substantial decrease in pH, as well as an increase in sulfide concentration.

The substantial change in alpha diversity is corroborated by a high turnover of ASVs between the layers and timepoints (Fig. 4, Additional file 1: Figure S8). The top layer is well separated from the deeper layers. The communities at 25 cm water depth experienced the largest turnover, i.e. change in community structure, and showed a loss in diversity during the experiment that seemed to have recovered at the last time point (Fig. 4). The communities of all three deep layers (10–35 cm) had a similar community structure at the beginning of the experiment. Interestingly, during the course of the experiment the community structure of each layer followed a different trajectory, yet at the end converged again. The trajectories of layer 2–4 indicate that the bloom shifted the microbial communities in these layers to an alternative stable state.

**Fig. 6:**
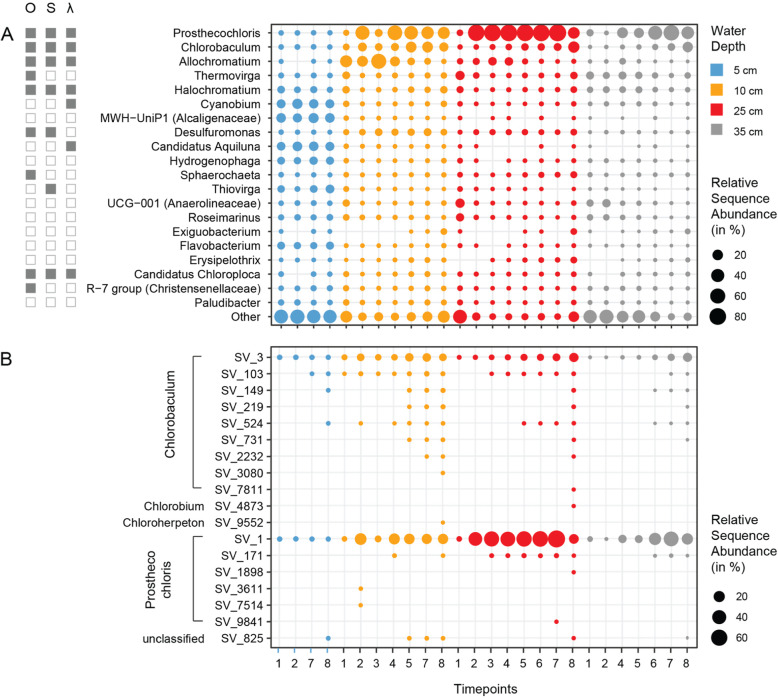
Bacterial community composition on genus level. **a** Relative sequence abundance of genera found in different depth layers (colors) and timepoints (x-axis). Relative sequence abundances were averaged across triplicates, due to the high similarity of all three experiments. Clades that are anaerobic (O), involved in the sulfur cycle (S), or phototrophic (ƛ) are indicated by full squares. **b** Relative sequence abundance of amplicon sequence variants (ASVs) within the order *Chlorobiales*. The graph shows average values of the three replicate experiments for clarity. The replicate experiments were very similar (see SI Additional file 1: Figure S9 and S10)

The taxonomic composition was assessed at all phylogenetic levels (Additional file 1: Figure S9B). We observed a total of 73 bacterial phyla. The surface community (5 cm) remained relatively unchanged throughout the experiment and was dominated by *Proteobacteria*, *Chlorobi*, *Cyanobacteria* and *Actinobacteria*. The communities in the deeper oxygen poor and sulfide rich zones (10–35 cm) were more dynamic, being dominated by *Bacteroidetes*, *Proteobacteria*, *Firmicutes*, and *Chloroflexi*. In general, taxonomic diversity was highest in the deepest layer (35 cm). The observed change in microbial diversity was accompanied by a change in community composition. Within a few days, there was a substantial increase in the abundance of *Chlorobi*, which comprised more than 75 % of the community at that time. This increase persisted for nine days, but levelled off at the end of the experiment. The datasets of all layers and timepoints were dominated by ASVs affiliating with phototrophic organisms, as shown by relative sequence abundances on genus level (Fig. 6a). Some phototrophs occurred in all layers at similar relative sequence abundances, such as *Halochromatium* and “*Candidatus* Chloroploca”. The stable surface layer harbored *Cyanobium* and “*Candidatus* Aquiluna”, which decreased in the deeper layers. The upper layer of the bloom showed an increased relative sequence abundance of *Allochromatium,* the lower bloom layer was dominated by *Prosthecochloris* and *Chlorobaculum* (Fig. 6a, b, Additional file 1: Figure S10). In addition to phototrophs the bloom layers were enriched with sulfur-reducing *Desulfuromonas sp.* as well as *Exiguobacterium sp.* (Fig. 6a, Additional file 1: Figure S11). The layer above the bloom was slightly enriched with sulfur-oxidizing *Thiovirga sp.* and the layer below the bloom with *Erypsipelothrix sp.* Sulfate-reducing *Desulfobacteraceae* and *Desulfobulbaceae* were observed at low relative abundances in all layers (Additional file 1: Figure S9B).

Interestingly, almost all *Prosthecochloris* affiliated reads belonged to a single sequence variant, while ASV diversity affiliated with the closely related *Chlorobaculum* increased over time (Fig. 6b, Additional file 1: Figure S10). The relative sequence abundance of *Chlorobiales* was highest at 25 cm depth, coinciding with the microbial bloom layer that was richest in biomass (Fig. 2) and had the highest cell numbers (Additional file 1: Figure S4). *Chlorobiales* ASVs accounted for > 25 % of reads in our dataset. To identify the phylogeny of ASV affiliating with *Chlorobiales*, we placed the representative sequence of each ASV on a reference tree of known *Chlorobiales*. The most abundant *Chlorobiales* ASV (ASV_1) affiliated with the genus *Prosthecochloris*, specifically with the monophyletic clade of *Prosthecochloris vibrioformis* (Additional file 1: Figure S12), followed by an ASV (ASV_2) affiliating with *Chlorobaculum*. Together, these two ASVs account for > 97 % of the *Chlorobiales* reads. In general, we found a high number of unclassified lineages. The 20 most abundant ASVs accounted for about 50 % of all sequences, twelve of those belonged to unclassified genera or families (Additional file 1: Figure S9B). The novelty was especially high within the *Chromatiaceae* where five ASVs, that ranked among the “top 20”, belonged to an unclassified genus.

### Metagenomics-derived insights into *Chlorobiales* populations

We calculated the index of replication (iRep) [42] of the *Prosthecochloris* and *Chlorobaculum* populations based on the metagenome-assembled genomes (MAGs) that were recovered from the community metagenomes of two replicate experiments (Replicates A, E) and the enrichment culture (SK) at timepoint 7. Both populations were replicating rapidly. *Prosthecochloris* (bin10) had an iRep value of 3.7 (r^2^ = 0.90, sample 7A3), which indicates that on average every cell had 2.5 replication events at the time of sampling. *Chlorobaculum* (bin 6) had iRep values of 2.5 (r^2^ = 0.95, sample 7E3) and 2.8 (r^2^ = 0.95, sample 7K3), indicating that on average every cell had ~ 1.5 replication events. Both MAGs contained genes involved in oxidative sulfur metabolism including Dsr, SoxYZ (Additional file 1: Figure S17), Sqr and Fcc. Bin 6 also contained SoxXAB while Bin 10 contained PhsA. Components of assimilatory sulfate reduction (CysND and Cys) were also found in both MAGs. Genes for bacteriochlorophyll biosynthesis (BchEMU) were found in both MAGs. Bd-type oxidases (CydAB) were present in both MAGs, while heme-copper oxygen reductases were only found in Bin 6 including several cytochrome c oxidases (COX10, CyoABCDE and III) (Additional file 1: Table S4).

Bin 6 (*Chlorobaculum sp.*) and bin 10 (*Prosthecochloris sp.*) contained CRISPR arrays denoted as either type I (cas3) or III (cas10) CRISPR systems [43] (Additional file 1: Figure S18, S19). CRISPR predictions revealed three direct repeat sequences in both MAGs of 30, 35 and 35 bp in length for Bin 6 and 37, 32, and 33 for the Bin 10 (Additional file 1: Table S5). None of the spacers were shared by the closest reference and representative genomes or matched sequences in the CRISPR database [44]. However, a highly similar CRISPR array and direct repeat sequence were found between Bin 6 and *Chlorobaculum parvum* NCBI8327 with 60 % *cas* genes similarity (Additional file 1: Figure S18). The metagenomes of all experiments, as well as of the GSB enrichment culture contained high relative sequence abundances of viruses affiliating with *Myoviridae* (Additional file 1: Figure S20).

## Discussion

In this study, we created depressions in the organic matter layer of Trunk River to mimick disturbances of the layer that naturally occur at this site. We performed triplicate experiments that resulted in very similar physicochemical gradients and patterns of community structure enabling us to reliably study microbial community succession in a natural setting. The observed slight variations between replicate sites were likely due to small differences in the organic matter composition and distance to the lagoon inflow, or caused by weather, animals, and sampling. Disturbing the organic matter layer at our experimental sites (A-, E-, and K-hole) released trapped sulfide and caused the rapid establishment of steep physicochemical gradients as well as the development of a bloom of sulfide-oxidizing phototrophs. We monitored the microbial community assembly and succession, highlight the ecological niches of key populations and indicate syntrophic interactions between phototrophs and sulfur reducers.

### Sulfur cycling in the phototrophic bloom

**Fig. 7:**
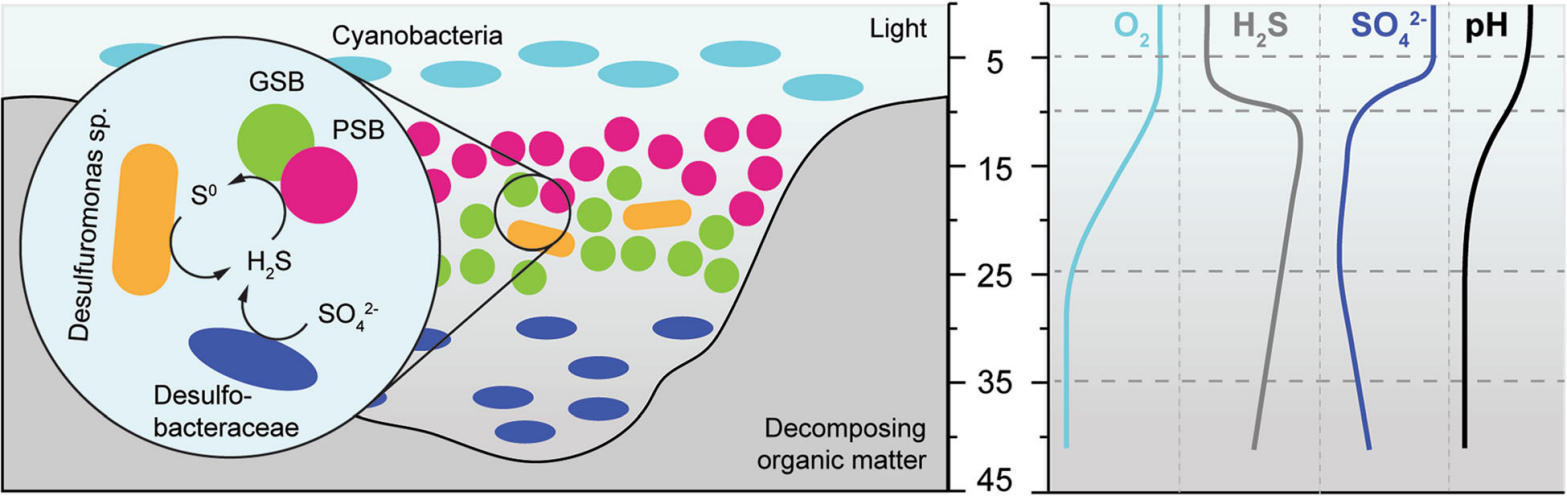
Schematic overview of the phototrophic bloom showing relevant sulfur-cycling and phototrophic populations, sulfur compounds, and chemical gradients, as well as potential syntrophic interactions between green sulfur bacteria (GSB), purple sulfur bacteria (PSB) and *Deltaproteobacteria*. Depth is given in cm

Sulfate concentrations in the bottom layers decreased substantially within the first days and were lowest in the bloom layer at 25 cm depth, where sulfate was almost entirely depleted. We found sulfate-reducers affiliating with *Desulfobacteraceae* and *Desulfobulbaceae* in the hypoxic layers of the bloom (Additional file 1: Figure S9B) likely producing sulfide using either hydrogen or organic acids, e.g. acetate (Additional file 1: Figure S6) released from fermented organic matter. The sulfide concentrations were highest at the upper boundary of the bloom at 10 cm water depth after the system stabilized around day six (Fig. 2). This is unexpected since reduced sulfur species, especially hydrogen sulfide, are the electron donor for the green and purple phototrophs and thus should have been depleted in these layers. At the same time, we found an increased relative abundance of sulfur-reducing *Desulfuromonas sp.* in the bloom layers, peaking at around 15 % relative sequence abundance. *Desulfuromonas sp.* are known to live in freshwater ecosystems and reduce elemental sulfur to sulfide [45–47], which in turn can be reused by the sulfide-oxidizing phototrophs. Our findings suggest that the initially present sulfide was released from the sediment but was likely replenished by sulfate reducers from sulfate, as well as by sulfur reducers from sulfur. Sulfide (and thiosulfate) are oxidized to elemental sulfur by the anoxygenic phototrophs and hence the potential sulfur reduction by *Desulfuromonas sp.* indicates a syntrophic short sulfur cycle carried out by these organisms (Fig. 7). A similar synergistic interaction was suggested to occur in Lake Cadagno between sulfur disproportionating *Desulfocapsa thiozymogenes* and purple sulfur bacteria affiliating with *Lamprocystis* [48]. At early timepoints the microbial suspension was beige and opaque, indicating the presence of large amounts of elemental sulfur in the sample (Additional file 1: Figure S2). Later the samples turned yellow, likely due to an increase in phototrophic organisms and their photopigments (Figs. 2, 3 and 6), but also the suspension became translucent again (Additional file 1: Figure S2). This suggests that after a few days *Desulfuromonas sp.* reduced the elemental sulfur (possibly present as polysulfides) that was produced by the anoxygenic phototrophs and initially accumulated in the suspension. An observation that merits future research. Such a syntrophic sulfur cycle represents a positive feedback that could explain the abundance of sulfide in the bloom as well as the very rapid growth of the sulfur-oxidizing phototrophs. The involved phototophs and *Deltaproteobacteria* could even form tight aggregates similar to *Chlorochromatium aggregatum* [49], to efficiently use the sulfur intermediate.

### Assembly and coexistence of phototrophic microorganisms

The multispecies phototrophic bloom (fondly termed “microbial lemonade”, Fig. 1c) formed around two to four days post-disturbance and was fully established by day six. The bloom contained lineages from multiple phyla but was dominated by green and purple sulfur bacteria. The color of the bloom slightly shifted from beige at early timepoints to yellow-orange at mid timepoints to yellow-green at late timepoints (Additional file 1: Figure S2), likely due to the relative influence of the photopigments of green and purple sulfur bacteria. The change in bacteriochlorophylls is reflected by the pigment spectra collected at the different timepoints (Fig. 3). The opacity and color of the suspension, especially at the beginning of the experiment, is likely influenced by the presence of polysulfides that are produced abiotically [50], as well as biotically by purple and green sulfur bacteria due to their lack of soxCD genes [51].

Interestingly, the sequencing data suggest that especially the lower layer of the bloom was dominated by an apparently clonal population of green sulfur bacteria affiliated with *Prosthecochloris vibrioformis*. The green sulfur bacteria are sulfur-oxidizing, strictly anaerobic, obligate photoautotrophs [52]. Yet, based on oxygen measurements, the Trunk River GSB populations tolerated relatively high oxygen concentrations of around 30 μM, but up to 80 μM (Fig. 2). The low concentration of dissolved oxygen at 25 cm depth combined with sulfide, salinity, and low light created an optimal habitat for *Prosthecochloris sp*. The observed community turnover (Fig. 5) indicates that communities in the layers 2–4 shifted from one stable state at the beginning of the experiment (timepoint 1) to an alternative stable state at the end of the experiment (timepoint 8). It appears that PSB (*Allochromatium sp.*) played a key role in stable state one, while the community of stable state two was equally dominated by both GSB populations (*Prosthecochloris sp.* and *Chlorobium sp.*). The change of relative abundances of phototrophs over the course of the experiment seems to be responsible for the pronounced community turnover, because together these few clades made up the majority of sequence reads. *Chlorobiales* have a high tolerance towards sulfide, and a higher affinity for sulfide than *Chromatiales* [53]. Together with their metabolic potential to cope with oxygen and their efficient growth at low light conditions [54] these capabilities may have enabled GSB to outcompete PSBs at the end of the experiment leading to a community adapted to the changed conditions.

Despite the dominance of few populations the disturbance created a habitat with gradients of pH, salinity, light, oxygen, and sulfide that enabled the coexistence of multiple phototrophic clades from at least five different phyla (*Actinobacteria, Chlorobi, Chloroflexi, Cyanobacteria and Gammaproteobacteria*). Coexistence of multiple phototrophic lineages was observed before, especially in lakes [21, 55, 56]. The coexistence of organisms competing for the same energy source is due to the different absorption maxima of each clades’ photopigments (Fig. 3), as well as their need for different electron donors, and the varying salinity and oxygen tolerances of each clade. At Trunk River *P. vibrioformis* relatives were absent at 5 cm and present only in low abundance at 10 cm. The surface layer (5 cm depth) was inhabited by oxygenic phototrophic *Cyanobacteria* affiliating with *Cyanobium*, while the upper layer of the bloom (10 cm depth) was dominated by purple sulfur bacteria of the order *Chromatiales* (Fig. 6). Because *Prosthecochloris* are adapted to low light conditions [57] and respond to different wavelengths of light than *Cyanobacteria* and photosynthetic *Proteobacteria* [58, 59], they thrived at depths of 25 cm, where they out-competed other phototrophs. *Prosthecochloris* have been previously observed in many marine and saline habitats, such as the Black Sea [60], Baltic Sea, Sippewissett Salt Marsh, and Badwater basin [52]. They are considered to belong to a specialized phylogenetic lineage of green sulfur bacteria adapted for marine and salt water ecosystems. Blooms of *P. vibrioformis* have been previously observed in stratified lakes, where they dominate the community at a specific depth [61], sometimes forming clonal blooms [62].

The phototrophs in the pelagic bloom were layered analogous to the phototrophs in benthic mats in the nearby Sippewissett Salt Marsh [63–65] and elsewhere [66, 67]. The disturbance experiment apparently created transient pelagic ecosystems with niches resembling those in benthic phototrophic mats. The bloom slowly collapsed after about two weeks and the water column seemed to return to almost its original state (Fig. 4). We did not observe a shift from phototrophic to chemotrophic sulfur oxidation after the phototrophic bloom [21].

### New species of green sulfur bacteria and possible viral predation

In a previous study based on 16S rRNA gene libraries, Imhoff and colleagues proposed the existence of several uncultivated GSB species in Sippewissett Salt Marsh and other estuaries [52]. The authors provide evidence that several GSB clades harbor species that have defied isolation, among those are species in the genera *Chlorobaculum* and *Prosthecochloris*. We have strong evidence that we found at least two of these uncultured species based on MAGs of a *Chlorobaculum* species (Bin 6, Additional file 1: Figure S13, S15) and a *Prosthecochloris* species (Bin 10, Additional file 1: Figure S13, S16). Both MAGs cluster sufficiently far away from the closest cultured isolate (Additional file 1: Figure S12, S14) and have average nucleotide identity (ANI) values of < 90 to their respective closest cultured isolate.

The MAGs of the phototrophic populations represented by bin 6 and 10 encoded for enzymes performing sulfide and thiosulfate oxidation. All known GSB contain the sulfide:quinone oxidoreductase (SQR) and the dissimilatory sulfite reductase (DSR) system (the latter is missing only in *Chloroherpeton thalassium*) that oxidize sulfide to sulfite [68]. Bin 6 and 10 featured the SQR and DSR systems as well. Additionally, bin 10 contained PhsA encoding for a thiosulfate reductase that may further oxidize sulfite to thiosulfate [69]. As in most GSB, bin 6 contained the genes SoxABXYZ coding for enzymes that oxidize thiosulfate to sulfate and polysulfides [70]. Bin 10 only contained SoxYZ (Additional file 1: Figure S17). The absence of SoxB genes has been identified in other non-thiosulfate oxidizing GSB such as the close relative *Prosthecochloris estuarii*, or in *Chlorobium limicola* DSM 245 and *Chlorobium luteolum* DSM 273 [71]. Both MAGs also contained the flavocytochrome c (FccB) involved in oxidative sulfur metabolism [68]. Bacteriochlorophyll synthesis genes were identical in both MAGs coding for pigments common to *Chlorobi*. In bin 6 we found complete operons encoding for cytochrome o oxidase (CyoABCDE) and cytochrome d oxidase (CydAB) [72]. The latter was found also in bin 10, indicating that both organisms have means to cope with oxygen stress. The presence of GSB at relatively high oxygen concentrations in Trunk River and their ability to perform anoxygenic photosynthesis at hypoxic conditions contrasts the general assumption that GSB are strict anaerobes in situ and in vitro [53, 73]. Heme-copper oxygen reductases similar to the ones we found in the *Chlorobi* MAGs have been found in other *Chlorobi* genomes including *Chlorobaculum parvum* (COX10, CyoABCDE, I, II, and III) and *Chlorobaculum limnaeum* (I, II, and III).

Both MAGs also contain CRISPR-Cas systems that are different from the closest cultured isolates (Additional file 1: Figure S18, S19). Our CRISPR results indicate that Trunk River populations are under viral predatory stress, affecting the abundance of bacterial blooms, and that host immunity is active in this ecosystem [74]. The unique CRISPR arrays indicate that closely related species may be infected by different viruses with species specificity [75]. However, some viral populations have been reported to have broad host ranges [76]. Divergent evolution or strain level microdiversity may also explain distinct CRISPR-Cas systems [77]. A lack of public databases containing viral sequences restricts the detection of viral-host interactions [78]. While Llorens-Marès et al. (2017) characterized a potential green sulfur bacteria viral infection, to date, phages infecting *Chlorobi* have not been reported. Our analyses suggest that viruses of the family *Myoviridae* played a role in the transient bloom (Additional file 1: Figure S20), and were possibly responsible for the blooms demise.

## Conclusions

In this study, we investigated phototrophic blooms that naturally occur in a brackish estuarine ecosystem to understand the underlying microbial and biogeochemical dynamics. Photosynthetically active radiation, the degree of anaerobiosis and nutrient supply are the main selecting factors in this stratified water body. The release of sulfide by heterotrophs and heterotrophic sulfate-reducers creates a habitat selecting for anoxygenic phototrophs, provided that enough light reaches the euxinic zone. The necessary light penetration occurs by disturbing or removing the dense covers of decaying seagrass, creating niches for phototrophic primary producers, sulfur oxidizers, as well as sulfur and sulfate reducers. We show that phototrophs belonging to five bacterial phyla spatially organized within the water column based on their light requirements and oxygen tolerance, forming a layered bloom, analogous to the layered communities in phototrophic microbial mats (Fig. 7). Our findings suggest the presence of a syntrophic sulfur cycle between anoxygenic phototrophs and sulfur reducers that could explain the rapid development of the bloom. We identified metagenome assembled genomes of two novel species of green sulfur bacteria, belonging to *Chlorobaculum* and *Prosthecochloris*. Contigs of viral sequences in the metagenomes suggest that *Myoviridae* viruses infect species within the *Chlorobiales*. This finding indicates a high degree of host-virus dynamics and a potential key regulating factor for the control of phototrophic blooms. In addition to genes coding for the multi-enzyme Sox complex, sulfide-quinone oxidoreductases, dissimilatory sulfite reductase and photopigment biosynthesis, the *Chlorobiales* MAGs also contained complete operons encoding for terminal oxidases, heme-copper oxygen reductases and cytochrome c and d oxidases. The activity of these oxidases may allow the organisms to thrive in the presence of oxygen. Future research addressing oxidase activity will tell whether *Chlorobiales* are as strictly anaerobic as is generally assumed. We consider the Trunk River lagoon an excellent model ecosystem to study the microbial community dynamics, syntrophy and ecophysiology in phototrophic bloom microbiomes in a natural setting.

## Methods

### Experimental setup and sample collection

We used custom-made sampling poles for long-term environmental monitoring of the water column without disturbing the established gradients (Fig. 1b, c). The sampling poles were placed in three replicate depressions (A-hole, E-hole, and K-hole) that we dug into the thick layers of decaying organic matter (Fig. 1a). In each of the sites, a sampling pole was placed such that the inlets sampled water at 5 cm, 10 cm, 25 cm, and 35 cm depth below the water surface (Fig. 1b, c). Sampling poles were set up 1 day after the holes were created and sampling began 1 day after set up (2 days post disturbance), to allow disturbed sediment to settle. Samples were collected over a 15-day period during July–August 2015. For each sample, the first 50 ml were discarded, followed by collection of 100 ml of water in sterile tubes for further analyses. The tubes were transported on ice to the laboratory and stored at 4 °C. All sample collections were carried out between 4 pm and 6 pm.

### Enrichment culture

To enrich for GSB we used a defined saltwater medium (400 g/l NaCl, 60 g/l MgCl_2_*6H_2_O, 3 g/l CaCl_2_*2H_2_O, 10 g/l KCl) buffered at pH 7.2 with 5 mM MOPS. The medium contained 5 mM NH_4_Cl as N source, 1 mM K phosphate (pH 7.2) as P source, 70 mM NaHCO_3_ as C source, 10 mM Na_2_S_2_O_3_ as electron donor, 1 mM Na_2_S as reductant or electron donor, a multivitamin solution prepared at 1000× in 10 mM MOPS at pH 7.2, and a trace metal solution prepared at 1000× in 20 mM HCl. Saltwater base, MOPS, N- and P-source, and trace metals were autoclaved together in a Widdel sparging flask, cooled under a stream of N_2_/CO_2_ (80%:20%) gas. C-source, electron donors and vitamins were added from filter-sterilized stock solutions after cooling. The medium was inoculated with biomass removed from in situ enrichments of GSB grown on glass slides using a 770 nm monochromatic LED. After inoculation, the bottle was kept in dark for 2–4 h and then placed 5 cm away from a LED light source with the same specifications. After visible sign of growth – green coloration – the culture was filtered through 0.2 μm filter and used for DNA extraction, similar to other samples.

### Physicochemical measurements

In-situ measurements of pH, temperature, dissolved oxygen, oxidation reduction potential (ORP), and ion selective electrode (ISE) measurements were carried out with a multi-parameter probe equipped with a quarto probe (YSI Professional Series Model Pro). The probe was calibrated for pH with pH 4, 7, and 10 buffers and for dissolved oxygen using oxygen-saturated water and an anoxic solution of sodium ascorbate and sodium hydroxide. After each sample collection the probe was lowered into the water to each depth per site and after probe readings stabilized, the parameters were recorded.

To measure biomass and pigment spectra, up to 10 ml of the collected sample were filtered through a sterile Millipore filter (0.2 μm GTTP, 0.2 μm GNWP, or 0.22 μm GV). Filters were washed twice with ammonium acetate solutions with the same ionic strength as each depth. The filters were placed on aluminium foil, dried at 60 °C overnight and subsequently weighed (Additional file 1: Figure S3). A Spectral Evolution SR1900 spectrophotometer was used to measure the spectrum of the dried biomass on each filter with a scanning range of 350–1900 nm. The light source was a Dyonics 60 W lamp.

After sterile filtration, the filtrate was used to measure anion, cation, and organic acid concentrations using an ion chromatographer. The ion concentrations of samples were measured by diluting filtrate 1:10 with Millipore water to a total volume of 2 ml. The diluted samples were measured in triplicate using a ThermoFisher/Dionex ICS2100 equipped with an AS18 column using a 13 min, 33 mM NaOH isocratic program to measure anions and a CS12A column using a 13 min, 25 mM methane sulfonic acid isocratic program to measure cations. Samples for organic acid analysis were filtered through 0.2 μm filters and 900 μL of filtrate was added to 100 µL of 5 M H_2_SO_4_ to precipitate any compounds that might otherwise do so on the column. The samples were centrifuged and the upper portion was removed for HPLC analysis. Samples were analyzed on a BioRad Aminex HPX-87H column in isocratic elution mode with 5 mM sulfuric acid.

Iron concentration was quantified using the ferrozine assay [79]. 4.5 ml filtrate were added on site to 0.5 ml of 1 M HCl to prevent oxidation of any available Fe(III). For Fe(II), 50 μl filtrate was added to 50 μl of 1 M HCl and 100 μl of ferrozine (0.1 % [wt/vol] in 50 % ammonium acetate) was added. For total iron, 50 μl filtrate was added to 50 μl of 10 % hydroxylamine hydrochloride in 1 M HCl to reduce Fe(III) to Fe(II). Samples were added to 100 μl of ferrozine. All samples were incubated for 15 min and filtrate absorbances were read in triplicate at 560 nm using a Promega plate reader. Ferrous ammonium sulfate was used as standard.

Sulfide concentrations were quantified using the Cline assay [80]. 1.5 ml filtrate were added on site to 500 μl of zinc acetate solution (91 mM) to prevent oxidation of the sulfide. Cline reagent (N, N-dimethyl-p-phenylenediamine sulfate, H_2_SO_4_, NH_4_Fe(SO_4_)_2_·12 H_2_O) was added, the samples were incubated in the dark for 30 min and absorbance was read at 665 nm. A table with all physicochemical and biomass measurements is publicly available at PANGAEA (https://doi.pangaea.de/10.1594/PANGAEA.900343).

### DNA extraction, library preparations, and sequencing

Within 2–6 h of sample collection, 50 ml sample was filtered using an autoclaved 0.2 μm polycarbonate filter (GTTP Millipore) and stored at − 20 °C. Each filter was cut with a sterile blade and extracted with the MoBio PowerFecal kit. We followed the protocol, but instead of bead beating, the samples were twice vortexed horizontally with the beads (10 min and 20 min with a 10 min pause). DNA concentration and purity were measured with Promega Qubit fluorometer and Nanodrop, respectively.

We prepared 16S rRNA gene amplicon libraries using V4-V5 fusion primers as previously described [81]. Briefly, the fusion primer contains TruSeq adapter sequences, barcodes, and the forward or reverse 16S rRNA gene primers. The forward and reverse 16S rRNA gene primers were 518F (CCAGCAGCYGCGGTAAN) and 926R (CCGTCAATTCNTTTRAGT). The PCR conditions were as follows: initial denaturation of 94 °C for 3 min, 30 cycles of denaturation at 94 °C for 30 s, annealing at 57 °C for 45 s, extension at 72 °C for 1 min, and final extension at 72 °C for 2 min. The libraries were cleaned using Agencourt Ampure XP beads, quantified using picogreen, pooled in equimolar ratios, and cleaned again using Agencourt Ampure XP beads a second time. The indexed libraries were then sequenced on the Illumina MiSeq PE250 platform.

DNA from 25 cm depth at timepoint 7 from each of the three replicate sites, as well as from a phototrophic enrichment culture were used to generate whole-genome shotgun metagenomic library. The DNA was sheared using Covaris sonicator, size selected for 500-600 bp using Pippin prep, and cleaned using Agencourt Ampure XP clean beads. The cleaned DNA was analyzed using Bioanalyzer DNA1000 chip and used to prepare metagenomic library using Nugen Ovation ultralow DR multiplex kit with manufacture supplied protocol. The libraries were then sequenced on Illumina MiSeq PE250 platform. All the sequencing was performed at the Keck facility at J. Bay Paul Center, Marine Biological Laboratory, Woods Hole, MA.

### Amplicon sequence data analyses

The amplicon data was demultiplexed in mothur v1.39.5 [82], followed by the trimming of 16S rRNA gene amplification primers using Cutadapt v1.16 [83] with default parameters. The primer-trimmed amplicon sequencing data was quality checked using DADA2 v1.9.0 R Package [84]. In DADA2, the reads were trimmed at the first instance of quality drop below 8, an expected error rate of 2, followed by trimming to 220 bp and 200 bp for forward and reverse reads. Any reads that matched PhiX or had an ambiguous base were removed. An error profile for the forward and reverse reads was generated using learnErrors function and then used to merge the forward and reverse reads using the mergePairs function. The merged reads were used to generate the amplicon sequence variants using makeSequenceTable function, which was then filtered for chimeras using removeBimeraDenovo function. The amplicon sequence variants were assigned taxonomy in DADA2 using Silva reference database v132 [85]. Community analyses were performed using a custom workflow based on R and the packages vegan, labdsv, tidyverse (stringr, dplyr, ggplot2), UpSetR and custom scripts [86–91]. Relative abundance of bacterial ASVs (amplicon sequence variants), Bray-Curtis dissimilarities, Nonmetric Multidimensional Scaling as well as analyses determining Singletons and percent shared ASVs are based on the unaltered Sample×ASV table as calculated by DADA2. The ASV × Sample table including taxonomy is available at PANGAEA (https://doi.pangaea.de/10.1594/PANGAEA.900354). To compare the diversity between samples using the number of observed species, Shannon index, Inverse Simpson diversity and Chao1 Richness [92] the ASV abundance tables were subsampled to account for unequal sampling effort using 31,682 randomly chosen sequences without replacement. For details refer to the R workflow available at the public database PANGAEA (https://doi.pangaea.de/10.1594/PANGAEA.900344).

### Metagenomic sequence data analyses

Quality control of the raw reads was performed using Preprocessing and Information of SEQuence data (PRINSEQ) to remove sequencing tags and sequences with mean quality score lower than 25, duplicates and ambiguous bases [93]. All runs combined provided a total of approximately 3.5 million 250 bp read pairs. All forward and reverse reads were placed together in one file and cross co-assembled with SPAdes using the --meta option [94]. Binning was performed using MetaBAT [95] and Anvi’o (v5.2) metagenomic workflow (CONCOCT) [96]. Completeness and contamination of bins was assessed using CheckM [97]. Assembled genomes that contained more than 90 % genome completeness, less than 5 % contamination, and sequences mainly from a single genus were further analyzed. This yielded two high quality bacterial metagenome-assembled genomes (MAGs): Bin 6 and Bin 10. Taxonomic composition for each bin was predicted using FOCUS [98]. Phylogenetic analysis including the identification of their closest phylogenetic neighbors was investigated using PATRIC Comprehensive Genome Analysis [99]. Gene prediction for MAGs was performed using prodigal (V2.60, −p meta). We searched for sulfur, terminal oxidases and chlorophyll pathways using Ghost-KOALA against the KEGG GENES database. The *Chlorobi* Bins 6 and 10 contained 2008 and 1938 predicted proteins, respectively. CRISPRCasFinder [100] and CRISPRone [101] were used to identify CRISPR repeat and spacer sequences. The quality checked reads from each sample were mapped to the MAGs, Bin 6 and Bin 10 using bowtie2 [102]. The mapped reads were then analyzed using iRep [42] to estimate replication events in Bin 6 and Bin 10. Unassembled sequences were processed on the MG-RAST platform version 4.0.3. Percent abundance of viral sequences was calculated from the RefSeq database using an e-value cutoff of 1e-5, a minimum identity cutoff of 60 %, and an alignment length minimum cutoff of 15 [103]. For details refer to the metagenome analyses workflow publicly accessible at HackMD (https://hackmd.io/tGZyCM9sSNmuorpHenQVNA).

## Additional file

**Additional file 1: **Supplementary Materials, Methods and Results. **Figure S1. **Natural blooms in Trunk River. **Figure S2. **Color and appearance of samples from all holes, depths, and timepoints. **Figure S3. **Filters that were used for biomass measurements and spectral analysis. **Figure S4. **Total cell count of three samples (A2, A7 and K7). **Figure S5. **Depth profile representation of chemical data presented in Fig. 2. **Figure S6. **Physicochemistry. Iron, nitrate, ammonium, acetate, Ca^2+^, and K^+^ measurements. **Figure S7. **Individual diversity indices of all samples. **Figure S8.** Trajectories of community structure in hole A, E and K. **Figure S9. **Relative sequence abundance of the 20 most abundant clades on phylum, class, order, family and genus level, as well as the 20 most sequence abundant ASVs (amplicon sequence variants). **Figure S10. **Relative sequence abundance of Chlorobiales ASVs. **Figure S11. **Relative change of ASV abundance between surface (V1) and deeper layers (V2-4). **Figure S12. **Chlorobiales phylogeny. **Figure S13. **Circular map of metagenome-assembled genomes (MAGs). **Figure S14. **Chlorobiales phylogenomics. **Figure S15. **Protein comparison of Bin 6. **Figure S16. ** Protein comparison of Bin 10. **Figure S17. **Genes involved in sulfur cycling**. Figure S18.** CRISPR arrays and cas genes predictions Bin 6. **Figure S19.** CRISPR arrays and cas genes predictions Bin 10. **Figure S20.** Relative sequence abundance of viral family-level clades. **Table S1.** Overview of sequencing output and diversity indices. **Table S2. **Genome statistics. **Table S3. **Average nucleotide identity (ANI) comparisons. **Table S4. **Oxidative phosphorylation and chlorophyll biosynthesis genes of Bin 6 and Bin 10. **Table S5. **CRISPR-Cas system information for each metagenome-assembled genome.

## Data Availability

The genomic datasets generated and analyzed during the current study are available on MG-RAST (Project Name: Trunk River, ID: 4837589.3 (sample SK), 4837590.3 (sample 7 K3), 4837591.3 (sample 7E3), 4837592.3 (sample 7A3)) and the metagenome-assembled genomes workflow is available on HackMD (https://hackmd.io/tGZyCM9sSNmuorpHenQVNA). The raw 16S rRNA gene amplicon data, the shotgun metagenomic data, the 16S rRNA gene clonal sequences, and the metagenome assembled genomes presented in this work are publicly archived in NCBI under Bioproject PRJNA530984 (https://www.ncbi.nlm.nih.gov/bioproject/530984). The contextual physicochemical datasets generated and analyzed during the current study are publicly available at PANGAEA under: 10.1594/PANGAEA.900343
