# Author Correction: Microbial community dynamics and coexistence in a sulfide-driven phototrophic bloom

**DOI:** 10.1186/s40793-023-00472-2

**Published:** 2023-03-30

**Authors:** Srijak Bhatnagar, Elise S. Cowley, Sebastian H. Kopf, Sherlynette Pérez Castro, Sean Kearney, Scott C. Dawson, Kurt Hanselmann, S. Emil Ruff

**Affiliations:** 1 Faculty of Science and Technology, Athabasca University, Athabasca, AB Canada; 2 Department of Bacteriology, University of Wisconsin-Madison, Madison, WI USA; 3 Department of Geological Sciences, University of Colorado, Boulder, CO USA; 4 Ecosystems Center and J. Bay Paul Center for Comparative Molecular Biology and Evolution, Marine Biological Laboratory, Woods Hole, MA USA; 5 Department of Biological Engineering, Massachusetts Institute of Technology, Cambridge, MA USA; 6 Department of Microbiology and Molecular Genetics, University of California, Davis, CA USA; 7 Department of Earth Sciences, ETH-Z, Zurich, Switzerland; 8 Microbiology Doctoral Training Program, University of Wisconsin-Madison, Madison, WI USA

**Correction **: **Environmental Microbiome (2020) 15:3** 10.1186/s40793-019-0348-0

The original article briefly discusses *Microviridae* viruses found in the metagenomes in the Abstract (Results), Results (Metagenome derived insights into Chlorobiales populations), Discussion (New species of green sulfur bacteria and possible viral predation), and Conclusions. Since the article’s publication it has come to light that the sequences associated with the *Microviridae* viruses belong to PhiX 174 (Genbank Accession Number NC_001422), a member of *Microviridae* family. These sequences were a contamination originating from the use of PhiX DNA as a control in Illumina sequencing platforms [1]. Hence, the authors would like to issue a correction to strike out any results and discussions associated with *Microviridae* virus(es). After removing PhiX 174-associated sequences from the metagenomic data, the most abundant viral sequences were affiliated with *Myoviridae*, a viral family which includes phages that are suspected to affect Chlorobi [2]. Figure S20 in Additional file 1 has been updated to reflect this change. This change does not affect any other results presented in the original article. The sequence data associated with this work was deposited in the NCBI SRA as raw sequence data and remains unaffected.

## Supplementary Information

**Additional file 1.** Supplementary Materials, Methods and Results. **Figure S1**.Natural blooms in Trunk River.** Figure S2**.Color and appearance ofsamples from all holes, depths, and timepoints.** Figure S3**.Filters thatwere used for biomass measurements and spectral analysis.** Figure S4**.Total cell count of three samples (A2, A7 and K7).** Figure S5**. Depthprofile representation of chemical data presented in Fig.2.** Figure S6.** Physicochemistry. Iron, nitrate, ammonium, acetate, Ca2+, and K+measurements. **Figure S7**. Individual diversity indices of all samples.** Figure S8.** Trajectories of community structure in hole A, E and K. **Figure S9**. Relative sequence abundance of the 20 most abundant clades onphylum, class, order, family and genus level, as well as the 20 mostsequence abundant ASVs (amplicon sequence variants).** Figure S10**.Relative sequence abundance of Chlorobiales ASVs.** Figure S11**.Relativechange of ASV abundance between surface (V1) and deeper layers (V2-4). **Figure S12.**Chlorobiales phylogeny. **Figure S13**. Circular map ofmetagenome-assembled genomes (MAGs). **Figure S14**. Chlorobiales phy-logenomics. **Figure S15.** Protein comparison of Bin 6.** Figure S16.**Pro-tein comparison of Bin 10. **Figure S17.** Genes involved in sulfurcycling.** Figure S18.** CRISPR arrays and cas genes predictions Bin 6.**Figure S19**. CRISPR arrays and cas genes predictions Bin 10.** Figure S20.** Relative sequence abundance of viral family-level clades. **Table S1.** Over-view of sequencing output and diversity indices. **Table S2.** Genome sta-tistics. **Table S3.** Average nucleotide identity (ANI) comparisons.** Table S4.** Oxidative phosphorylation and chlorophyll biosynthesis genes of Bin6 and Bin 10. **Table S5**. CRISPR-Cas system information for eachmetagenome-assembled genome.
